# The Antinociceptive Role of Nrf2 in Neuropathic Pain: From Mechanisms to Clinical Perspectives

**DOI:** 10.3390/pharmaceutics16081068

**Published:** 2024-08-15

**Authors:** Kestutis Petrikonis, Jurga Bernatoniene, Dalia M. Kopustinskiene, Roberto Casale, Sergio Davinelli, Luciano Saso

**Affiliations:** 1 Department of Neurology, Lithuanian University of Health Sciences, Eivenių Str. 2, LT-50009 Kaunas, Lithuania; kestutis.petrikonis@lsmu.lt; 2 Department of Drug Technology and Social Pharmacy, Faculty of Pharmacy, Medical Academy, Lithuanian University of Health Sciences, Sukileliu pr. 13, LT-50161 Kaunas, Lithuania; 3 Institute of Pharmaceutical Technologies, Faculty of Pharmacy, Medical Academy, Lithuanian University of Health Sciences, Sukileliu pr. 13, LT-50161 Kaunas, Lithuania; daliamarija.kopustinskiene@lsmu.lt; 4 Opusmedica Persons, Care & Research-NPO, 29121 Piacenza, Italy; robertocasale@opusmedica.org; 5 Department of Medicine and Health Sciences “V. Tiberio”, University of Molise, 86100 Campobasso, Italy; sergio.davinelli@unimol.it; 6 Department of Physiology and Pharmacology “Vittorio Erspamer”, La Sapienza University, 00185 Rome, Italy; luciano.saso@uniroma1.it

**Keywords:** neuropathic pain, Nrf2, oxidative stress, inflammation, mitochondria

## Abstract

Neuropathic pain, a chronic condition resulting from nerve injury or dysfunction, presents significant therapeutic challenges and is closely associated with oxidative stress and inflammation, both of which can lead to mitochondrial dysfunction. The nuclear factor erythroid 2-related factor 2 (Nrf2) pathway, a critical cellular defense mechanism against oxidative stress, has emerged as a promising target for neuropathic pain management. Nrf2 modulators enhance the expression of antioxidant and cytoprotective genes, thereby reducing oxidative damage, inflammation, and mitochondrial impairment. This review explores the antinociceptive effects of Nrf2, highlighting how pharmacological agents and natural compounds may be used as potential therapeutic strategies against neuropathic pain. Although preclinical studies demonstrate significant pain reduction and improved nerve function through Nrf2 activation, several clinical challenges need to be addressed. However, emerging clinical evidence suggests potential benefits of Nrf2 modulators in several conditions, such as diabetic neuropathy and multiple sclerosis. Future research should focus on further elucidating the molecular role of Nrf2 in neuropathic pain to optimize its modulation efficacy and maximize clinical utility.

## 1. Introduction

Neuropathic pain is a chronic pain condition resulting from nerve injury or malfunctioning, often described as burning, shooting, or tingling [1]. This type of pain is distinct from other forms of pain and can be chronic, severely affecting the quality of life.

Pain is typically categorized into three main types based on underlying mechanisms. Nociceptive pain results from direct injury to tissues, such as a cut or a fracture, and typically diminishes as the injury heals. It is usually associated with acute pain that is well localized and subsides with proper healing and treatment [2,3]. Neuropathic pain is caused by a lesion or disease of the somatosensory nervous system [4]. Unlike nociceptive pain, which results from tissue damage, neuropathic pain is caused by aberrant signaling within the nervous system itself. Common causes include nerve compression, trauma to the nervous system, diabetic neuropathy, and post-herpetic neuralgia [5]. Nociplastic pain is due to altered nociception, where there is no clear evidence of actual or threatened tissue damage causing the activation of peripheral nociceptors or of disease or lesion of the somatosensory system [6]. It is often seen in conditions such as fibromyalgia and irritable bowel syndrome [3,4].

In addition to these three main categories, some classifications also recognize a fourth type known as inflammatory pain, which is associated with the response of the immune system to tissue injury or infection [7,8]. Neuropathic pain often becomes chronic shortly after onset, persisting as a challenge for affected persons [9,10]. Patients typically endure long-lasting or sporadic spontaneous pain. Dysesthesia describes unusual, often painful sensations, whereas paresthesia is related to altered feelings such as numbness or tingling, which are generally not painful but might be discomforting [1]. These symptoms may co-occur, with patients reporting either increased or diminished sensitivity in the affected regions. Pain can be aggravated by movement or touch and might continue well beyond the healing of the initial injury [9,10]. The chronic nature of neuropathic pain results from the persistent sending of abnormal nerve signals to the brain [1]. It is related to peripheral and central sensitization, characterized by changes in ion channels, the activation of immune cells, the release of mediators from glial cells, and alterations in gene expression patterns, all contributing to the continuous experience of pain [11].

Neuropathic pain is a consequence of various disorders, including diabetes, shingles, chemotherapy, and physical trauma. This type of pain is difficult to treat due to its complex underlying mechanisms, which involve both peripheral and central nervous system changes [11]. The mechanisms involved in neuropathic pain are intricate and largely involve the complex interplay of immune response and inflammatory mediators within the central nervous system. One of the pivotal elements in this process is the role of pro-inflammatory cytokines, such as interleukin-1 beta (IL-1β). These cytokines are released by various types of cells within the spinal cord, specifically immune cells, microglia, and astroglia. IL-1β is particularly significant because it initiates and perpetuates inflammatory responses that are critical to the development and maintenance of neuropathic pain [12]. Additionally, the inflammatory process stimulates the expression of cyclo-oxygenase-2 (COX-2) in the affected tissues. COX-2 is an enzyme that plays a vital role in the inflammatory pathway by converting arachidonic acid into prostaglandins, particularly prostaglandin E2 (PGE2) [13]. Prostaglandins are potent inflammatory mediators that further contribute to the sensitization of the central nervous system. They amplify pain signals not only by affecting the nerve cells directly but also by promoting inflammation, which leads to additional tissue damage and pain [14]. The prolonged presence of these inflammatory mediators can lead to a perpetuating cycle of pain and inflammation, making neuropathic pain particularly resistant to typical pain management strategies [15,16,17].

Neuropathic pain is also linked to oxidative stress, which can induce mitochondrial dysfunction. Reactive oxygen species (ROS) can activate various signaling pathways, including those involved in pain transmission and modulation. Moreover, mitochondrial damage can contribute to the development of neuropathic pain through the generation of ROS and the disruption of calcium buffering [18]

Recently, nuclear factor erythroid 2-related factor 2 (Nrf2) has gained significant attention for its central role in managing neuropathic pain [19,20,21]. By enhancing the antioxidant response, Nrf2 mitigates the oxidative stress associated with nerve damage, reducing the hyperexcitability of neurons that often leads to pain [22]. In addition, Nrf2 plays a crucial role in maintaining the structural and functional integrity of mitochondria, promoting mitochondrial quality control, influencing mitochondrial biogenesis, and maintaining redox homeostasis [23]. It also regulates inflammation by promoting the expression of anti-inflammatory mediators, thereby stabilizing the cellular environment and diminishing pain-related inflammation [19]. Thus, Nrf2 may be a prominent player in reducing the neuropathic pain [21].

## 2. Overview of the Nrf2 Pathway

Nrf2 is a transcription factor crucial in the modulation of cellular defense mechanisms against toxic and oxidative stress by regulating genes essential for the oxidative stress response and detoxification of drugs [20,24,25]. The activation of Nrf2 enhances the ability of cells to survive in the presence of stressful conditions, such as chemical carcinogens or inflammatory stimuli [24,26]. Beyond its role in antioxidant defense, Nrf2 also regulates genes vital for the regulation of cellular metabolism and inflammation processes [24,25].

**Figure 1:**
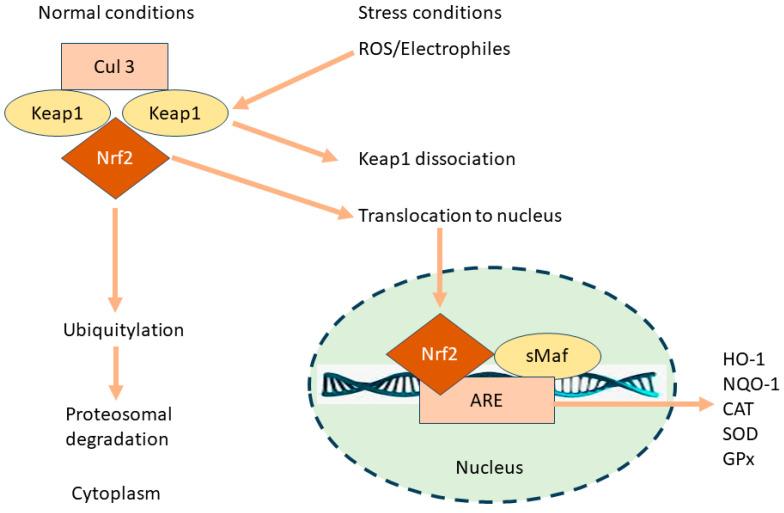
Nrf2 effects under normal and stressful conditions. Keap1—Kelch-like ECH-associated protein 1, Cul 3—Cullin3, ARE—Antioxidant Response Element, sMaf—small Maf proteins, NQO-1—NAD(P)H quinone oxidoreductase 1, HO-1—heme-oxygenase-1, CAT—catalase, SOD—superoxide dismutase, GPx—glutathione peroxidase.

Under normal physiological conditions, Nrf2 is bound in the cytoplasm by Keap1 (Kelch-like ECH-associated protein 1), which acts as a sensor for oxidative stress and electrophiles (Figure 1) [24,27].

Keap1 functions not only as an anchor but also as a substrate adaptor for Cullin 3-based E3 ubiquitin ligase [28], which ubiquitinates Nrf2, targeting it for proteasomal degradation [29]. This binding keeps Nrf2 levels low in the absence of stress. Upon exposure to oxidative stress or electrophilic agents, modifications occur in Keap1, typically through the alteration of specific cysteine residues [28]. These modifications disrupt the Keap1–Nrf2 interaction, leading to the stabilization and accumulation of Nrf2 [29]. Nrf2 then translocates from the cytoplasm into the nucleus [30]. This migration is facilitated by the escape from Keap1-mediated degradation and the exposure of nuclear localization signals on Nrf2. In the nucleus, Nrf2 binds to the Antioxidant Response Element (ARE) in the promoter regions of target genes [31,32]. ARE is a specific sequence in DNA that acts as a regulatory enhancer element promoting the transcription of various antioxidant and cytoprotective genes [32]. Nrf2 typically forms heterodimers with small Maf proteins for effective binding to ARE [33]. Binding to ARE leads to the subsequent transcription of target genes [32]. These genes encode for a wide array of antioxidant and phase II detoxification enzymes, such as glutathione S-transferase, heme oxygenase-1 (HO-1), and NAD(P)H: quinone oxidoreductase 1 [24]. Upregulated HO-1 breaks down heme into carbon monoxide (CO), bilirubin, and free iron [34]. CO functions as an inhibitor of the nuclear factor-kappa B (NF-κB) pathway, resulting in reduced expression of pro-inflammatory cytokines. Bilirubin also serves as an antioxidant. Additionally, HO-1 directly suppresses pro-inflammatory cytokines and stimulates anti-inflammatory cytokines, thereby helping to regulate and balance the inflammatory response [34,35].

Other genes activated by Nrf2 include those involved in the synthesis and regeneration of glutathione (GSH), a critical cellular antioxidant, and various stress response proteins [20]. The increased expression of these detoxifying and antioxidant proteins enhances the ability of cells to neutralize reactive oxygen species (ROS) and other harmful compounds. Such response helps in restoring cellular redox homeostasis, protecting the cell from oxidative damage, and promoting cell survival under stress conditions [20]. Under conditions where Nrf2 is either underactive or overactive, cellular susceptibility to oxidative damage changes significantly. For instance, insufficient Nrf2 activity can leave cells vulnerable to oxidative stress, contributing to the progression of various diseases [30,36]. On the other hand, excessive Nrf2 activity, often due to genetic mutations or cancerous alterations, can lead to an abnormal resistance against cellular apoptosis, aiding the survival of malignant cells [32,37].

The p62 protein, which is important for autophagy, interacts with Keap1 to disrupt its inhibition of Nrf2, leading to Nrf2 activation and enhanced antioxidant gene expression. This mechanism is critical under oxidative stress or when autophagy is impaired, as p62 sequesters Keap1, facilitating nuclear translocation of Nrf2. Similarly, the PI3K/Akt/GSK-3 (phosphoinositide 3-kinase, protein kinase B (Akt), and glycogen synthase kinase-3) pathway regulates Nrf2 activity through Akt-mediated inhibition of glycogen synthase kinase-3 β (GSK-3β), stabilizing Nrf2 and promoting cellular survival under stress [38]. Additionally, retinoid X receptor alpha (RXRα) enhances the transcriptional activity of Nrf2 by interacting directly with gene promoters, boosting the cellular defense against oxidative damage. Collectively, these pathways underscore complex regulatory mechanisms impacting the role of Nrf2 in cellular protection against oxidative stress and related pathologies [35,38].

The therapeutic potential of modulating the Nrf2 pathway is broad, with implications for treating a range of oxidative stress-related conditions [36]. Pharmacological activators of Nrf2, such as sulforaphane found in broccoli and other cruciferous vegetables [39,40], or synthetic compounds like oltipraz [41], have shown promise in upregulating antioxidant defenses in experimental models. These activators could potentially mitigate the effects of chronic oxidative stress seen in conditions like Alzheimer’s disease, diabetes, and rheumatoid arthritis. Conversely, strategies to inhibit Nrf2 are being explored as potential treatments for cancers where Nrf2 contributes to chemoresistance and the enhanced survival of cancer cells [26,36,37]. Additionally, Nrf2 upregulation has been observed in non-cancer treatments involving toxic drugs, indicating its role as an adaptive mechanism against drug-induced damage across various treatment scenarios [24].

The overactivation or constitutive activation of the Nrf2/ARE antioxidant system can contribute to a range of diseases, including multidrug-resistant cancers, autoimmune disorders, cardiovascular diseases, neurodegenerative conditions, fibrosis, and metabolic issues, by promoting tumor cell survival and resistance, disrupting immune homeostasis, and negatively impacting metabolic and neuronal functions [42,43,44,45]. The chronic activation of Nrf2 has been linked to the progression and aggressiveness of certain cancers [43,46]. Elevated Nrf2 levels can promote tumor cell survival, growth, and resistance to chemotherapy by enhancing the antioxidant capacity of cancer cells, making them less susceptible to oxidative stress-induced cell death [42]. Increased Nrf2 activity in cancer cells can lead to resistance against chemotherapeutic agents and radiation therapy. This resistance arises because Nrf2 enhances the expression of detoxifying enzymes and efflux pumps, which reduces the efficacy of cancer treatments [47]. Overactive Nrf2 signaling can contribute to the development of fibrosis in organs such as the liver and lungs by promoting the expression of fibrotic mediators and extracellular matrix components [48]. While Nrf2 activation has anti-inflammatory effects, inappropriate or prolonged activation can disrupt immune homeostasis. This disruption might exacerbate autoimmune diseases or chronic inflammatory conditions by interfering with the normal immune response [44]. Excessive Nrf2 activation may negatively impact lipid metabolism, leading to fatty liver disease, and may also affect glucose metabolism, contributing to insulin resistance and the development of type 2 diabetes [49].

## 3. Modulation of Oxidative Stress in Neuropathic Pain by Nrf2 Activation

Oxidative stress plays a crucial role in the initiation and maintenance of neuropathic pain, making it a significant target for therapeutic intervention [21]. Oxidative stress refers to the imbalance between the production of ROS or reactive nitrogen species (RNS) and the capability of antioxidant defense to detoxify these reactive products. ROS include free radicals such as superoxide anion (O_2_^−^), hydrogen peroxide (H_2_O_2_), and highly reactive hydroxyl radicals, which can damage cells by altering lipids, proteins, and DNA [50]. In neuropathic pain, oxidative stress is both a by-product of nerve injury and a factor that exacerbates pain [21]. When nerves are damaged, they respond with an inflammatory process that involves the activation of glial cells, such as microglia and astrocytes in the central nervous system [51,52]. This activation leads to the production of pro-inflammatory cytokines and chemokines, which further stimulate the production of ROS [53]. Moreover, the disrupted mitochondrial function commonly observed in damaged neurons enhances ROS production, thereby aggravating oxidative stress and contributing to a cycle of nerve damage and pain sensation [21]. The mechanisms through which oxidative stress contributes to neuropathic pain involve direct neuronal damage and the modulation of pain-related signaling pathways [51,52]. Oxidative stress can enhance central sensitization—increased functioning of neurons and circuits in nociceptive pathways caused by elevated membrane excitability and synaptic efficacy, as well as reduced inhibition, which is a characteristic feature of the development of neuropathic pain [52]. ROS can potentiate this process by activating pain pathways in the spinal cord, leading to increased pain perception [21]. Oxidative stress could also trigger and amplify neuroinflammatory responses, which can further sensitize peripheral nerves [34,35]. This is partly achieved through the activation of NF-κB, a key transcription factor that regulates the expression of genes involved in inflammation and immune responses [54]. By modifying proteins, lipids, and nucleic acids, ROS could alter the function and survival of both neuronal and non-neuronal cells in the nervous system [50]. These biochemical alterations can disrupt cellular homeostasis and impair the ability of neurons to transmit normal sensory signals [21].

**Figure 2:**
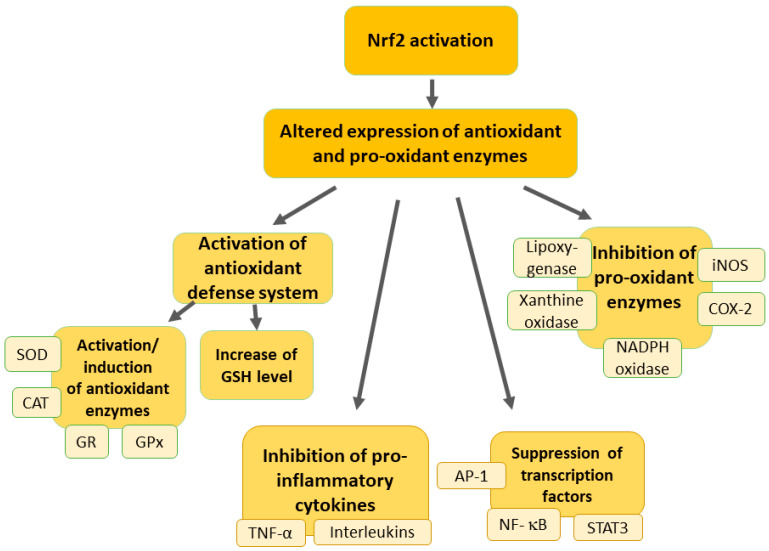
Indirect antioxidant effects of Nrf2 activation in the cells. TNF—tumor necrosis factor, AP-1—activator protein 1, NF-κB—nuclear factor kappa-light-chain-enhancer of activated B cells, STAT3—signal transducer and activator 3, COX-2—cyclooxygenase-2, iNOS—inducible nitric oxide synthase, SOD—superoxide dismutase, CAT—catalase, GPx—glutathione peroxidase, GR—glutathione reductase, GSH—glutathione.

Activation of the Nrf2 pathway can upregulate the expression of several antioxidant and cytoprotective genes, providing a robust cellular defense against oxidative stress (Figure 2) [21].

Nrf2 functions as a transcription factor that regulates the expression of genes responsible for antioxidant and cytoprotective responses, which is crucial for modulating the cellular environment and reducing oxidative stress. This, in turn, influences the excitability of neurons and the overall pain experience [55]. At the synapse level, Nrf2 plays several roles in modifying pain transmission. First, it enhances antioxidant defenses by upregulating enzymes such as heme oxygenase-1 (HO-1), NAD(P)H quinone oxidoreductase 1 (NQO-1), superoxide dismutase (SOD), glutathione cysteine ligase, glutathione S-transferases, catalase, and others [50,52]. This reduction in oxidative stress decreases the excitability of nociceptors and the release of pro-nociceptive substances. Additionally, Nrf2 reduces the production of pro-inflammatory cytokines and chemokines, thereby diminishing neuroinflammation and subsequent pain signaling [56,57,58]. Furthermore, Nrf2 affects synaptic plasticity by modulating ion channels involved in pain transmission, such as voltage-gated sodium and calcium channels, altering neuronal excitability [59,60,61]. By reducing oxidative stress, Nrf2 also decreases the abnormal release of excitatory neurotransmitters like glutamate at the synapse, thus attenuating the amplification of pain signals [62]. Nrf2 provides neuroprotection by maintaining the integrity of pain pathways and preventing the pathological changes associated with chronic pain [63]. It achieves this by protecting neurons from damage induced by oxidative stress and neuroinflammation. Moreover, Nrf2 acts on glial cells, such as astrocytes and microglia, to reduce their activation and the release of pro-inflammatory mediators, thereby modulating synaptic transmission and pain perception [64]. Thus, Nrf2 modulates pain transmission by acting on various types of neurons in the pain pathway through its role in reducing oxidative stress and inflammation. At the synapse level, it regulates antioxidant defenses, influences synaptic plasticity, and provides neuroprotection, ultimately affecting neuronal excitability and neurotransmitter release and influencing the overall experience of pain [55].

Nrf2 expression has been observed in the spinal cord, dorsal root ganglion, and sciatic nerve of rats [21]. HO-1, a major effector of Nrf2 activation, has been shown to exert significant antinociceptive effects. Studies have demonstrated that inducing HO-1 can markedly inhibit pain, as seen when cobalt protoporphyrin (CoPP, a HO-1 inducer) significantly reduced formalin-induced inflammatory pain, an effect reversed by tin protoporphyrin (SnPP, an HO-1 inhibitor) [65]. Notably, HO-1 was upregulated in the spinal cord in neuropathic pain models, with repeated CoPP administration or over-expression of HO-1 via lentivirus significantly reducing pain behaviors, potentially inhibiting glial-mediated neuroinflammation [66]. Diosmetin, a flavonoid extracted from citrus fruits in a concentration-dependent manner (10–100 mg/kg), alleviated neuropathic pain in a chronic nociceptive pain model in mice by upregulating HO-1 and Nrf2 [67]. Mitsugumin53 (MG53), a protein involved in cellular membrane repair, improved neuropathic pain, neuroinflammation, and oxidative stress via activation of the Nrf2/HO-1 signaling pathway in the rat spinal cord of a chronic constriction injury model [68]. In another study, the cerebrospinal fluid from patients with trigeminal neuralgia accumulated ROS, several of which directly activated the pain-transducing channel transient receptor potential ankyrin 1 (TRPA1) in trigeminal neuron cell cultures. Stimulating the NRF2 antioxidant transcriptional network could reduce pain via the inhibition of TRPA1, in part by reversing the underlying oxidative stress [63]. An angiotensin II Type 1 receptor blockade via losartan attenuated the neuropathological changes in the spinal cords of diabetic rats with modulation of the Nrf2/HO-1 pathway [69]. Electroacupuncture treatment on male Sprague–Dawley rats with chronic constriction injury protected against neuropathic pain by inhibiting neuronal ferroptosis in the spinal cord dorsal horn, partially through the activation of Nrf2 signaling [70]. Curcumin treatment inhibited GSK-3β activation, increased Nrf2-mediated antioxidant responses, inhibited oxidative damage and inflammatory reactions, and alleviated oxaliplatin-induced neuropathic pain in mice. [71]

In a rat model of paclitaxel-induced neuropathic pain, Miao et al. reported a reduction in Nrf2, NQO-1, and superoxide dismutase 2 (SOD2) expressions in the dorsal root ganglion [72]. This reduction was countered by electroacupuncture, which restored the antioxidant defense, alleviating neuropathic pain [73]. Similarly, Sun et al. noted decreased Nrf2, NQO-1, and HO-1 in dorsal root ganglion from paclitaxel-treated rats, with improvements following treatment with alpha-lipoic acid [74]. Conversely, in a model of chronic constriction injury of the sciatic nerve, increased Nrf2 and HO-1 expressions were observed in the spinal cord, and treatment with sodium hydrosulfide alleviated pain symptoms by activating the Nrf2/HO-1 pathway [75]. Additionally, Zhou et al. indicated elevated Nrf2 and HO-1 in the spinal cord of rats with paclitaxel-induced neuropathic pain, with localization mainly in neurons [76]. These variable findings across different studies might be attributed to variations in animal models, the tissues examined, and the timing of sample collection, suggesting that Nrf2 may have distinct roles depending on the stage and tissue context.

In summary, many antinociceptive effects of Nrf2 are related to its role in regulating oxidative stress, providing a promising target for managing various pain conditions.

## 4. Protective Effects of Nrf2 against Inflammation in Neuropathic Pain

Prolonged chronic inflammation, marked by the deep infiltration of mononuclear immune cells such as monocytes, macrophages, lymphocytes, and plasma cells, alongside the increased production of inflammatory cytokines, is a fundamental aspect of many chronic conditions, including neuropathic pain [17,53,77]. This persistent inflammation is crucial in exacerbating their symptoms and severity. Typically, in the inflammatory response associated with neuropathic pain, immune cells migrate to the site of nerve injury, producing ROS that damage DNA and cellular structures and releasing various inflammatory mediators, such as cytokines, chemokines, and prostaglandins [17,53]. These mediators not only recruit more macrophages to the sites of inflammation but also activate several signal transduction pathways and transcription factors associated with inflammation, such as NF-κB, mitogen-activated protein kinase (MAPK), and Janus kinase-signal transducer and activator of transcription (JAK-STAT) pathways, intensifying the pain response [17,53,77].

Recent studies have highlighted the role of Nrf2 in modulating inflammatory processes and offering potential antinociceptive effects [34,35]. In neuropathic pain, the activation of Nrf2 reduces the levels of pro-inflammatory cytokines, such as tumor necrosis factor-alpha (TNF-α), and IL-1β, which are known to exacerbate pain signaling. Moreover, Nrf2 activation inhibits microglial activation and alleviates oxidative stress [34,35]. Oxidative stress can induce and sustain the inflammatory process, therefore the ability of Nrf2 to enhance cellular antioxidant defenses may reduce the oxidative stress associated with inflammation, thereby reducing pain. By reducing the levels of inflammatory cytokines and chemokines in injured areas, Nrf2 lowers the excitability of nociceptors and impairs the transmission of pain signals. The regulation of oxidative stress by Nrf2 may prevent nerve damage and dysfunction, common causes of neuropathic pain. This protection from oxidative damage reduces the likelihood of activating pain pathways [34,35].

As mentioned, exposure to oxidative stress can trigger the overproduction of pro-inflammatory cytokines through the activation of NF-κB, exacerbating oxidative stress in target cells and perpetuating a cycle of inflammation [78,79,80]. The activation of Nrf2 disrupts this cycle, while chemokines, a specific cytokine family, primarily attract immune cells like leukocytes and neutrophils to sites of inflammation [34,35]. Cell adhesion molecules (CAMs) such as ICAM-1 and VCAM-1, key members of the immunoglobulin superfamily, are surface proteins involved in cell recognition, activation, and signal transduction [81]. Nrf2 can inhibit the activity of VCAM-1 and regulate the expression of other adhesion molecules like E-selectin and VCAM-1 through the downstream gene HO-1, influencing leukocyte adhesion and recruitment [34,35]. Matrix metalloproteinases (MMPs) are prevalent in the extracellular matrix and play roles in both physiological and pathological processes, including cell proliferation, migration, differentiation, wound healing, angiogenesis, apoptosis, and tumor metastasis [82]. In inflammatory conditions, MMP regulation is directly influenced by the Nrf2 pathway or indirectly via the NF-κB pathway, which is also modulated by Nrf2 [34,35]. Nrf2 also downregulates pro-inflammatory COX-2 and inducible nitric oxide synthase (iNOS) genes [83,84], as well as modulates inflammasome activity [85,86,87].

Bardoxolone methyl alleviated chemotherapy-induced neuropathic pain in rats treated by intraperitoneally injecting paclitaxel via a decrease in inflammatory markers due to the activation of Nrf2 in the dorsal root ganglia [88]. The effects of omaveloxone were tested on mechanical allodynia in a chronic constriction injury (CCI) rat model, resulting in reduced neuronal apoptosis and glial cell activation due to the increase in Nrf2 expression and decrease in the inflammatory response [89]. Betulinic acid (3–30 mg/kg for 8 days) decreased neuropathic pain induced by CCI of the sciatic nerve in mice by activating the Nrf2/HO-1 signaling pathway, inhibiting glial cell activation, and downregulating the expression levels of pro-inflammatory cytokines [90]. The effects of luteolin on mood disorders such as anxiety and depression, triggered by chronic neuropathic pain, were investigated by examining the impact on oxidative stress, neurotrophic factors, and neuroinflammation in a rodent model of CCI. Luteolin treatment (10–50 mg for 21 days) alleviated mood disorders by modulating oxidative stress markers, neurotrophic levels, and inflammatory mediators such as IL-1β, IL-18, IL-6, and TNF-α in the rat hippocampus and prefrontal cortex [91]. Resolvin D1/N-formyl peptide receptor 2 diminished paclitaxel-induced neuropathic pain through the activation of the IL-10/Nrf2/HO-1 pathway in mice [92]. Moreover, ajugarin-I (5 mg/kg) significantly reduced vincristine-induced neuropathic pain behaviors in mice, such as hyperalgesia and allodynia, and reversed the histological damage in the sciatic nerve, spinal cord, and brain [93]. It alleviated oxidative stress and inflammation by modulating Nrf2/NF-κB signaling, reduced apoptosis through the Bcl-2/Bax and caspase-3 pathways, and enhanced antioxidant capacity while decreasing inflammatory cytokines [93]. The neuroprotective effects of berberine (50 or 100 mg/kg orally for 10 days) were studied in a proteasome inhibitor bortezomib-induced peripheral neuropathy model in Sprague–Dawley rats, resulting in decreased oxidative stress and inflammation markers and increased antioxidant levels. Berberine effectively mitigated bortezomib-induced neuropathic changes by suppressing pro-inflammatory cytokines and enhancing protective gene expression in the sciatic nerve and spinal cord [94]. Diabetic encephalopathy was addressed in a study examining the neuroprotective effects of the *Securidaca inappendiculata* polyphenol-rich extract (SiPE) in diabetic rats. The study found that SiPE treatment significantly improved behavioral deficits, reduced neuropathic pain, and alleviated depressive-like behaviors by modulating oxidative stress, inflammation, p38 MAPK, and Nrf2, ultimately enhancing antioxidant enzyme activity in the brain [95]. The effects of the α_2_-adrenergic receptor agonist dexmedetomidine on neuropathic pain were investigated in a CCI rat model, focusing on its interaction with the Keap1-Nrf2-HO-1 pathway. The results demonstrated that dexmedetomidine treatment reduced pain severity, downregulated Keap1, and upregulated Nrf2 and HO-1 in the spinal cord, effectively alleviating inflammation, apoptosis, and oxidative stress, thereby mitigating neuropathic pain [96]. Also, dexmedetomidine alleviated neuropathic pain in a CCI rat model by suppressing the inflammasome through the activation of Nrf2, leading to reduced spinal cord injury, apoptosis, and inflammation [97]. Thus, the activation of Nrf2 could reduce inflammation by suppressing pro-inflammatory cytokines and enhancing antioxidant defenses, thereby alleviating pain and potentially preventing further nerve damage [34,35].

## 5. Mitochondrial Function and Neuropathic Pain: The Role of Nrf2

Neurons, due to their complex functions and extensive signaling activities, have significantly higher energy demands compared to other cell types, and that energy is supplied by mitochondria [98]. Adenosine triphosphate (ATP) is crucial not only for maintaining the neuronal membrane potential but also for restoring it following an action potential, ensuring rapid and efficient signal transmission across neural networks [99]. In addition to their role in energy production, mitochondria function as vital regulators of intracellular calcium (Ca^2+^) levels [100]. They act as Ca^2+^ reservoirs, managing and moderating the concentration of this important signaling ion within the cellular environment, which is critical for numerous neuronal functions including neurotransmitter release and synaptic plasticity [101].

The equilibrium between mitochondrial dynamics, including fission and fusion processes, and mitochondrial turnover, which encompasses both biogenesis and mitophagy, is crucial for sustaining healthy mitochondrial function [102]. Additionally, maintaining calcium levels and ROS balance within mitochondria is essential for their optimal operation [103]. Disruption in any of these processes can lead to mitochondrial dysfunction, characterized by an overproduction of ROS [104]. ROS are not only produced in mitochondria but also in several other cellular compartments, including the cell membrane, cytoplasm, endoplasmic reticulum, peroxisomes, and Golgi apparatus [105]. However, mitochondria are the primary source of ROS, where these reactive molecules are generated as byproducts of the electron transport chain during aerobic respiration [104,105]. Excessive ROS production, especially when not adequately neutralized by antioxidant systems, can damage cellular proteins, lipids, and DNA, leading to cellular injury and a cascade of molecular events that may result in chronic disorders [104]. Furthermore, the peroxidation of cardiolipin, a phospholipid in the inner mitochondrial membrane essential for maintaining cristae structure and stabilizing cytochrome c for oxidative phosphorylation, increases ROS and RNS generation, causes mitochondrial swelling, triggers cytochrome c release, and impairs oxidative phosphorylation [106,107]. Thus, the regulation of mitochondrial health and the control of ROS production are vital for preventing cellular damage and maintaining overall cellular and body health [104].

Mitochondrial dysfunction is a key contributor to the development of neuropathic pain [102]. The mitochondria, essential for energy production and cellular metabolism, can become impaired due to injury or disease, leading to increased production of ROS and subsequent oxidative stress, which in turn can activate pain pathways and exacerbate nerve damage [102,108,109]. Oxidative stress activates pain pathways by producing excessive ROS-damaging neurons and -sensitizing receptors, modulating ion channels, stimulating the production of pro-inflammatory mediators, causing peripheral nerve injury, and activating cellular signaling pathways like MAPK and NF-κB [55]. These effects collectively enhance inflammation and pain signal transmission, leading to increased pain sensitivity.

Mitochondrial dysfunction contributes to hyperalgesia by disrupting energy production, increasing ROS production, altering calcium homeostasis, triggering apoptosis, and activating inflammatory pathways [102]. These disruptions enhance pain signaling, promote inflammation, and lead to the loss of inhibitory neurons, all of which heighten sensitivity to pain [102,108,109]. Modulating mitochondrial functions in sensory neurons has been shown to reduce hyperalgesia in pre-clinical models of both neuropathic and inflammatory pain [18,110,111,112,113,114]. In these studies, alterations in mitochondrial activity led to significant decreases in pain sensitivity. Furthermore, ultrastructural abnormalities in mitochondria—an increased number of swollen and vacuolated mitochondria—were observed mostly in peripheral nerve sensory axons, with several cases in dorsal root ganglia and even in Schwann cells in the cases of diabetic neuropathy and chemotherapy-induced neuropathic pain models as well as chronic constriction injury of the sciatic nerve models [18]. Furthermore, transient inflammation induced hyperalgesic priming in sensory neurons, increasing the expression of the ATP synthase c subunit lysine N-methyltransferase (ATPSc-KMT) and causing mitochondrial disturbances, thus impairing pain resolution; however, inhibiting mitochondrial respiration, knocking down ATPS_C_-KMT, or supplementing the affected metabolite could restore pain resolution and prevent chronic pain development [115].

**Figure 3:**
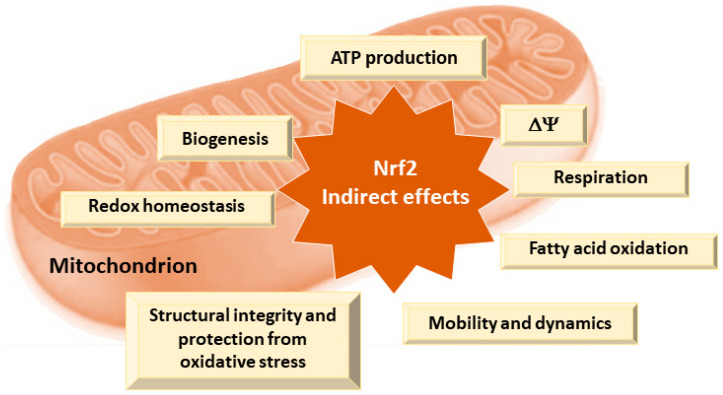
Indirect Nrf2 effects on mitochondrial functions. ΔΨ—mitochondrial membrane potential.

Nrf2 is one of the main regulators of cellular homeostasis that helps to maintain cellular bioenergetics by controlling substrate availability for mitochondrial respiration and enhancing mitochondrial biogenesis and efficiency by regulating genes involved in antioxidant defense and mitochondrial quality control [109]. Nrf2 reduces the effects of ROS produced during oxidative phosphorylation [108,109]. In conditions where uncoupling of oxidative phosphorylation occurs, such as in the presence of mild mitochondrial stress, Nrf2 is activated and promotes the expression of antioxidant and cytoprotective genes to restore redox balance and protect cellular components from oxidative damage [108]. This process, known as mitohormesis, involves a beneficial response to low levels of stress that improves cellular function and resilience [102,108]. Numerous aspects of mitochondrial physiology and homeostasis are reliant on Nrf2 activity (Figure 3) [109].

Nrf2 plays a critical role in maintaining cellular redox homeostasis by regulating the production of ROS through the biosynthesis, utilization, and regeneration of GSH, thioredoxin, and NADPH [109,116]. Its activation induces the expression of mitochondrial antioxidant proteins such as GR, GPx, thioredoxin reductase 2, peroxiredoxin 3, peroxiredoxin 5, and SOD2, which help mitigate oxidative stress [109]. Additionally, Nrf2 regulates the redox activity of metal ions, particularly iron homeostasis, by targeting genes involved in heme biosynthesis and the expression of ferritin and ferroportin genes [117].

Most mitochondrial proteins are encoded by nuclear genes, giving the nucleus significant control over mitochondrial biogenesis and function [118,119]. Recent research, however, highlights the regulatory role of bioactive mitochondria-derived peptides (MDPs) encoded by short open reading frames (sORFs) within the mitochondrial genome [120,121]. Mitochondrial biogenesis requires tight coordination between mitochondrial and nuclear transcription factors, with markers including the mtDNA/nDNA ratio and the expression of genes such as peroxisome proliferator-activated receptor gamma coactivator 1-alpha, mitochondrial transcription factor A, and Nrf1 [122,123]. Nrf2 enhances this process by promoting genes involved in nucleotide synthesis and metabolism through PI3K/Akt signaling. Additionally, AMPK, an energy sensor, regulates mitochondrial processes and circadian rhythms and activates Nrf2 by inhibiting glycogen synthase kinase-3 beta GSK3β. Furthermore, PGAM5, by interacting with Nrf2 and Keap1, facilitates the regulation of antioxidant gene expression, with inhibitors like LFHP-1c enhancing Nrf2 activity [109].

The functional connection between Nrf2 and the mitochondrial network is mediated by interactions with mitochondrial proteins and the regulation of ROS balance [124]. Retrograde signaling mechanisms, including the mitochondrial unfolded protein response (UPRmt) and damage-associated molecular patterns (DAMPs), play a crucial role in cellular stress responses [125]. The mitochondrial MOTS-c peptide, encoded by the mitochondrial genome, translocates to the nucleus under metabolic stress and, in coordination with AMPK and SIRT1, regulates the expression of ARE-containing genes by interacting with Nrf2, acting as its cofactor [126,127,128]. The overexpression of MOTS-c enhances cellular protection against stress from glucose or serum deprivation, highlighting its significant role in nuclear gene response to metabolic stress [126,128].

Thus, by enhancing mitochondrial resilience, Nrf2 activation helps maintain energy production and cellular homeostasis [109], further indirectly contributing to pain relief in neuropathic conditions. This makes Nrf2 activation not only a means to counteract the immediate effects of mitochondrial dysfunction but also a potential long-term strategy to restore cellular function and reduce neuropathic pain.

## 6. Nrf2 Modulation in Neuropathic Pain: Pharmacological and Alternative Strategies

Recent systematic reviews have reported the analgesic effects of Nrf2 inducers in various animal pain models, as well as their toxicity and side effects [19,21,22]. In this section, we discuss the effects of traditional and alternative therapies for neuropathic pain, focusing on their mechanisms of action and their potential to modulate the Nrf2 signaling pathway.

Common pharmacotherapies for neuropathic pain include anticonvulsants like gabapentin and pregabalin, tricyclic antidepressants such as amitriptyline, and serotonin-norepinephrine reuptake inhibitors (SNRIs) like duloxetine. Topical agents, including lidocaine and capsaicin creams, are also used. Non-steroidal anti-inflammatory drugs (NSAIDs) and selective COX-2 inhibitors help manage inflammation-related pain. For specific cases, N-methyl-D-aspartate (NMDA) receptor antagonists like ketamine are utilized, while opioids such as morphine and oxycodone are reserved for severe pain under strict medical supervision [1]. Recent studies have revealed that despite well-established mechanisms of action of common drugs used for neuropathic pain, they might also potentially modulate the Nrf2 pathway (Table 1).

The gabapentinoids gabapentin and pregabalin bind to the α2δ subunit of voltage-gated calcium channels, inhibiting the release of excitatory neurotransmitters [140,141]. However, several investigations have shown that gabapentin could modulate the Nrf-2/HO-1 pathway in sepsis-induced acute kidney injury model in rats [129]. Also, higher expression of Nrf2 was observed in the brains of pregabalin-treated mice in a streptozotocin-induced diabetic mice model, which suggested immunomodulatory and anti-inflammatory effects of pregabalin [130]. One of the primary mechanisms of action of tricyclic antidepressants is the inhibition of serotonin and norepinephrine reuptake in presynaptic terminals, increasing their availability in the synapse, which enhances neurotransmitter activity and modulates pain signaling pathways to reduce pain perception, with additional effects arising from NMDA receptor modulation and ion channel blockade [142,143]. Furthermore, amitriptyline suppressed TNF-α-induced MAPK phosphorylation as well as the activity of NF-κB in HUVEC cells, suggesting that amitriptyline reduces endothelial inflammation, consequently improving vascular endothelial function [131]. SNRIs are medications that reduce pain signals by increasing serotonin and norepinephrine levels through the inhibition of their reuptake [144]. However, antidepressants could regulate the Nrf2 pathway in the prefrontal cortex (PFC) and hippocampus in male Wistar rats. While this pathway is involved in mitigating oxidative/nitrosative damage in the PFC, contributing to the therapeutic effects of antidepressants, it appears that Nrf2 does not play a role in the effects of chronic mild stress (CMS) in the hippocampus [132]. SNRI duloxetine alleviated asthma symptoms through anti-inflammatory and antioxidative responses regulated by the PI3K/AKT/mTOR and Nrf2/HO-1 signaling pathways [133] and protected human neuroblastoma cells from oxidative stress-induced cell death through the Akt/Nrf-2/HO-1 pathway [134].

Lidocaine, an amide-type local anesthetic, blocks sodium channels to inhibit pain signal transmission and modulates NMDA receptors to reduce chronic pain and central sensitization, enhancing its effectiveness in treating neuropathic pain [145,146,147]. However, lidocaine could attenuate hypoxia/reoxygenation-induced inflammation, apoptosis, and ferroptosis in lung epithelial cells by regulating the p38 MAPK pathway [135] and could relieve murine allergic rhinitis by regulating the NF-κB and p38 MAPK pathways [136]. Capsaicin desensitizes nociceptive nerve fibers, which transmit pain signals to the brain. Applied topically, it binds to TRPV1 channels, initially causing a burning sensation, and then reduces pain sensitivity by depleting substance P, a neurotransmitter involved in pain transmission [148]. However, capsaicin could reduce protein aggregation, improve mitochondrial function, decrease ROS generation, and activate the Nrf2-mediated pathway to enhance antioxidant defenses and inhibit inflammation in neurodegenerative diseases [149].

In neuropathic pain, overactive NMDA receptors in the spinal cord dorsal horn increase pain sensitivity and central sensitization. NMDA antagonists block these receptors, reducing excitatory pain signal transmission to the central nervous system, thereby alleviating neuropathic pain and preventing chronic pain states [150,151,152]. Although there are no direct data on whether ketamine can modulate Nrf2-mediated pathways in neuropathic pain models, there is evidence that ketamine can diminish inflammation and oxidating stress acting via Nrf2. Ketamine could diminish airway inflammation by inducing inflammatory cell-mediated pathways in apoptosis and activating the Nrf2 pathway in a mixed-granulocytic murine asthma model [137]. Furthermore, ketamine ameliorated oxidative stress-induced apoptosis in an experimental traumatic brain injury via the Nrf2 pathway [138]. Moreover, ketamine decreased the release of high-mobility group box 1, a late-phase cytokine of sepsis, in lipopolysaccharide-stimulated macrophages via activation of the Nrf2/HO-1 pathway and NF-κB suppression [139].

Neuropathic pain, resulting from nerve damage or dysfunction, often challenges effective and well-tolerated treatment due to the limited relief and significant side effects associated with traditional analgesics like opioids and NSAIDs [153]. Investigating new drug targets and emerging pharmacotherapies, including the repurposing of existing drugs, shows promise for enhancing pain treatment options and patient outcomes [154]. Furthermore, these compounds could also exert their effects via Nrf2 (Table 2).

Ambroxol has the potential to manage neuropathic pain by inhibiting Nav1.8 voltage-gated sodium channels, thereby reducing nociceptive neuron excitability and pain signal transmission [177,178]. Moreover, ambroxol enhanced Nrf2 activity and upregulated HO-1 and catalase to strengthen antioxidant defenses against oxidative damage, while also inactivating NF-κB signaling and reducing pro-inflammatory mediators IL-6 and TNF-α in an acetic acid rat model of ulcerative colitis [155]. Furthermore, ambroxol decreased the protein expression of nitrotyrosine, iNOS, NF-κB, and the MAPK signaling cascade with a concomitant increase in the expression of Nrf-2 and SOD-1 in RAW 264.7 cells and skin tissues in a psoriasis-like skin inflammation model [156].

Cannabidiol (CBD) is a promising candidate for treating neuropathic pain due to its analgesic and anti-inflammatory properties. It interacts with the endocannabinoid system, modulating pain and inflammation through cannabinoid receptors CB1 and CB2 [159]. CBD demonstrated protective effects against neuroinflammation in rats by enhancing Nrf2 signaling and reducing oxidative stress [157]. CBD modulated the farnesoid X receptor (FXR)/Nrf2 pathway and altered the CB1/CB2 receptor ratio in a rat model of gentamicin-induced kidney injury [158]. CBD was able to regulate the Nrf2 system by interacting with the NF-κB pathway under oxidative stress [179]. CBD-restored paclitaxel reduced the expression of p-AMPK, SIRT1, NRF2, HO1, SOD2, and catalase while increasing the expression of PI3K, p-AKT, p-38 MAP kinase, Bax, TGF-β, NLRP3 inflammasome, and caspase 3 in the dorsal root ganglion homogenates of mice [159].

Bromelain is primarily used for digestion but its potential properties to reduce pro-inflammatory cytokines, nitrate levels, and iNOS expression in the sciatic nerve and its ability to modulate biological processes have sparked interest in its use for neuropathic pain [155]. Bromelain alleviated neuropathic pain in a chronic constriction injury-induced model in Wistar rats by enhancing the activities of nuclear transcription factors Nrf-1 and Nrf-2, which activated the antioxidant defense system, thereby reducing neuronal stress and structural disorganization [160].

Melatonin regulates circadian rhythms and exhibits antioxidant properties, protecting against lipid peroxidation, inflammation, and tumor growth while promoting apoptosis and mitochondrial function [180,181]. It crosses cell membranes and the blood–brain barrier, allowing it to modulate pain signaling pathways through interactions with opioid, adrenergic, and cannabinoid receptors, as well as MT1 and MT2 melatonin receptors, reducing cyclic AMP formation and nociception [182]. Melatonin modulates pain signaling in the CNS by interacting with opioid, adrenergic, and cannabinoid receptors: it enhances the analgesic effects of endogenous opioids and opioid medications by increasing receptor expression and endorphin release, reduces pain sensitivity by inhibiting norepinephrine release through adrenergic receptors, and exerts analgesic and anti-inflammatory effects by enhancing the activation of cannabinoid receptors, thereby contributing to comprehensive pain relief and management [182]. Melatonin also suppresses pro-inflammatory cytokines like IL-1β, TNF-α, and NOS, and acts as an antioxidant, minimizing neuronal damage and inflammation [182]. Melatonin could protect neurons from damage by activating the Nrf2-ARE signaling pathway [161,162,163]. Melatonin significantly increased the Nrf2 and HO-1 level in experimental colitis in rats [161]. Melatonin suppressed lipoprotein-associated phospholipase A2 expression and atherosclerosis processes by inhibiting macrophage ferroptosis and partially activating the Nrf2 pathway in mice macrophages [162]. Melatonin ameliorated multiorgan injuries induced by severe acute pancreatitis in mice by regulating the Nrf2 signaling pathway, and these effects were absent in Nrf2-knockout mice [163].

N-acetyl-L-cysteine (NAC) has been studied for its potential therapeutic effects on neuropathic pain due to its antioxidant and anti-inflammatory properties [183,184]. As a precursor of GSH, NAC enhances the antioxidant defenses, reducing oxidative stress and inflammation, which are key contributors to nerve damage and neuropathic pain [183,184]. Additionally, NAC modulates neurotransmitter activity and influences excitatory and inhibitory signaling in the central nervous system, including interactions with glutamatergic and GABAergic systems [61], and the activity of transient receptor potential melastatin-like 2 (TRPM2) channels [61]. NAC also increased the expression of miR-141 and activated Keap1/Nrf2 signaling in male Sprague–Dawley rats [164].

As alternative therapies, acetyl-L-carnitine, palmitoylethanolamide, natural antioxidants/anti-inflammatory compounds (resveratrol, curcumin, and sulforaphane), and non-coding RNAs could help in the management of neuropathic pain [185,186,187]. Acetyl-L-carnitine may manage neuropathic pain by modulating neurotransmitters like glutamate and GABA in the spinal cord dorsal horn, promoting nerve regeneration, exerting antioxidant and anti-inflammatory effects, and influencing synaptic plasticity [188,189,190]. Acetyl-L-carnitine administration to rats under hypoxic conditions induced mitochondrial biogenesis through a PGC-1α and Nrf-1 pathway regulated by an ERK-Nrf2 mechanism and improved hippocampal neuron bioenergetics by buffering calcium into nonfunctional mitochondria, reducing excitotoxicity [165]. Moreover, it prevented hypobaric hypoxia-induced spatial memory impairment through extracellular-related kinase-mediated Nrf2 phosphorylation [166]. Palmitoylethanolamide, an endogenous fatty acid, modulated inflammation and pain by activating cannabinoid receptors and inhibiting inflammatory mediators, demonstrating significant analgesic effects in neuropathic pain [191]. Furthermore, it was able to relieve spinal cord injury in rats by inhibiting inflammatory responses and oxidative stress, which may involve a mechanism associated with the activation of Nrf2/HO-1 via the Raf-1/MEK/ERK pathway [167]. Resveratrol, found in grapes and berries [192,193], and curcumin, derived from turmeric [194,195], both exhibited anti-inflammatory and analgesic properties in pre-clinical studies, modulating pain and inflammation pathways in neuropathic pain. Resveratrol exerted its analgesic effect by modulating pivotal signaling pathways, including PI3K/Akt/mTOR, TNFR1/NF-κB, MAPKs, and Nrf2 [168]. Resveratrol could alleviate the severity of diabetic neuropathy by protecting peripheral nerves from apoptosis by inhibiting the NF-κB pathway and increasing Nrf2 expression [169]. Early resveratrol administration prevented NF-κB, TNF-α, and activating transcription factor 3 (ATF3) upregulation while increasing the expression of Nrf2, NQO-1, HO-1, and the redox-sensitive deacetylase SIRT1 in an oxaliplatin-induced mechanical and thermal allodynia model [170]. In an oxaliplatin-induced neuropathic pain model in mice, curcumin inhibited the inflammasome-mediated inflammatory response, enhanced Nrf2/GPx4-mediated antioxidant defenses, and reduced mitochondrial oxidative stress, while also binding to GSK-3β through four covalent bonds to decrease its activity in the spinal cord [71]. Furthermore, the synergistic application of curcumin and catalase promoted the regulation of the Nrf2/HO-1 signaling pathway by curcumin [171]. Sulforaphane is a natural Nrf2 pathway activator that could reduce inflammation and oxidative stress, thus helping to alleviate neuropathic pain [19]. Sulforaphane could mitigate intervertebral disc degeneration by reducing endoplasmic reticulum stress in nucleus pulposus cells through the activation of the Nrf-2/HO-1 pathway [172]. Sulforaphane intake suppressed exercise-induced oxidative stress and muscle damage and reduced delayed onset muscle soreness in humans after eccentric exercise by activating the Nrf2 pathway and increasing NQO-1 expression [173]. Inflammation and oxidative stress can lead to the development of tolerance to opioids. By reducing inflammation and oxidative stress, sulforaphane can enhance the efficacy of morphine [174]. Sulforaphane alleviated hyperalgesia and enhanced the analgesic potency of morphine in rats with cancer-induced bone pain [174]. In a rat model of sciatic nerve endometriosis, sulforaphane alleviated pain by inducing COX2 and iNOS suppression and upregulated Keap1 and Nrf2, thereby inhibiting inflammation [175]. Sulforaphane treatment normalized oxidative stress by activating Nrf2/HO-1 signaling, reduced microglial activation, and decreased JNK, ERK1/2, and p-38 phosphorylation induced by a sciatic nerve injury in the spinal cord, hippocampus, and prefrontal cortex. Additionally, sulforaphane treatment enhanced the antiallodynic effects of morphine in mice with a sciatic nerve injury [176].

In neuropathic pain, specific miRNAs have been identified as crucial regulators of pain-related pathways, modulating genes involved in neuronal sensitization, synaptic plasticity, and inflammatory responses. By targeting these molecules, miRNAs can significantly influence the development and persistence of neuropathic pain [196]. miRNA-497 could regulate the ubiquitin carboxyl-terminal hydrolase 15 (USP15)/Nrf2/Glucose-6-phosphate dehydrogenase (G6PD) axis. miRNA-497 was downregulated in the dorsal root ganglion neurons of rats with streptozotocin-induced diabetic neuropathic pain, alleviating pain by inhibiting USP15, which otherwise promotes Nrf2 degradation and reduces G6PD expression [197]. Also, miRNA-155 silencing reduced sciatic nerve injury in diabetic peripheral neuropathy [198].

There may also be alternative therapeutic effects on Nrf2-mediated inflammation and collateral damage, e.g., ozone therapy. Ozone activated Nrf2, and this activation triggered a complex cascade of events, which ultimately led to macrophage training and an improvement in their ability to operate a clearance of bacteria in the patient’s anatomical districts [199]. Also, ozone therapy reduced oxidative stress in post-chemotherapy polyneuropathy [200].

It has been demonstrated that depression is associated with brain inflammation, which activates microglial Toll-like receptors (TLR) and GSK3β. This activation shifts the balance between the pro-inflammatory NF-κB and the neuroprotective Nrf2 transcription factors [201]. Tricyclic antidepressants are thought to work by activating Gs protein-coupled (Gs-coupled) microglial receptors, increasing cAMP levels, and activating protein kinase A (PKA), which inhibits GSK3β. Experimental treatments, such as CB2 activation, opioid μ receptor agonists, 5HT2 agonists, valproate, ketamine, and vagus nerve stimulation, all inhibit GSK3β [201]. Therefore, it was proposed that screening for Nrf2 activation in microglial cells with TLR-activated GSK3β could lead to novel antidepressants with better efficacy [201].

Both neuropathic pain treatments and experimental antidepressant principles involve pathways that inhibit GSK3β, which subsequently shifts the balance towards the neuroprotective and anti-inflammatory effects mediated by Nrf2 activation. This suggests that targeting the Nrf2 pathway could be a promising strategy not only for developing more effective antidepressants but also for enhancing treatments for neuropathic pain. Thus, screening for Nrf2 activation in therapeutic research might lead to the discovery of novel, more efficacious drugs for neuropathic pain.

## 7. Challenges and Limitations of Antinociceptive Effects of Nrf2 Modulators in Neuropathic Pain

Recent research underscores the role of excessive ROS in the development of neuropathic pain, positioning Nrf2 as an important regulator of the antioxidant defenses [21,202]. Preclinical studies have demonstrated the potential of Nrf2 inducers to alleviate chronic inflammatory and neuropathic pain by mitigating ROS-related issues such as oxidative stress, mitochondrial dysfunction, and neuroinflammation [19]. Despite these promising findings, several limitations and unanswered questions remain.

Most current studies focus on the pain-relieving effects of Nrf2 inducers without fully exploring the underlying mechanisms. For example, Nrf2-mediated mitochondrial effects, which play a critical role in maintaining mitochondrial health and function, could be a significant factor in the analgesic activity of Nrf2 inducers [21]. Moreover, the lack of specificity in current Nrf2 inducers complicates their use [203]. These inducers are not exclusive activators of Nrf2, making it challenging to isolate their effects. Utilizing specific Nrf2 inducers and transgenic mouse models could provide clearer evidence of the role of Nrf2 in neuropathic pain [204]. Additionally, the complex nature of the oxidative stress in neuropathic pain suggests that using Nrf2 inducers alone might not be enough to obtain a complete response [19].

Furthermore, while increases in Nrf2 mRNA or protein levels have been observed following various interventions, these changes do not necessarily confirm that the analgesic effects are mediated through Nrf2 activation [19,21]. Studies using Nrf2 knockout mice [205] or specific Nrf2 inhibitors [36] are essential to establish a direct causal relationship between Nrf2 activation and pain relief.

Finally, despite the encouraging results from preclinical studies, there have been no clinical trials specifically investigating Nrf2 inducers for neuropathic pain management up to now. Given that some Nrf2 inducers have been safely used in clinical settings for other conditions, translating preclinical findings into clinical trials could be promising [41]. However, detailed research into the mechanisms of Nrf2 activators is necessary to ensure their efficacy and safety in treating neuropathic pain.

## 8. Clinical Perspectives of Nrf2 Modulators in Neuropathic Pain

Clinical evidence on the efficacy of Nrf2 modulators in neuropathic pain is still in its early stages. Despite numerous molecules being described as inducers and many in clinical development, only four—dimethyl fumarate (DMF), bardoxolone methyl, oltipraz, and sulforaphane—have been prominently featured in peer-reviewed literature, particularly in studies involving biomarker measures, with all four interacting with the Nrf2 signaling pathway through cysteine151 in Keap1 [41] (Table 3).

Fumaric acid esters, including DMF, are utilized for treating psoriasis and multiple sclerosis. The drug product Fumaderm^®^, a combination of DMF and mono ethylfumarate salts, was registered in 1994 for moderate to severe plaque psoriasis, while DMF alone (Skilarence^®^) also received EU approval for this condition. Due to its immunomodulatory properties, DMF was tested in large multicenter trials for relapsing–remitting multiple sclerosis, resulting in the approval of oral DMF (BG-12, Tecfidera^®^) by the FDA in 2013 and the EMA in 2014 [206]. DMF functions by preventing the degradation of Nrf2, thereby promoting the expression of genes that counteract oxidative stress and inflammation [206]. New analogs with improved bioavailability and efficacy are being developed, though DMF has been linked to adverse effects such as flushing, gastrointestinal issues, and occasionally persistent lymphopenia [41,206].

Another significant Nrf2 inducer is bardoxolone methyl, which has shown promising results in the treatment of chronic kidney disease (CKD) and other inflammatory conditions [207]. Clinical trials have demonstrated its potential to improve kidney function by reducing albuminuria and slowing the progression of CKD [41,207]. Additionally, bardoxolone methyl has been investigated for its anti-inflammatory properties in diseases such as pulmonary arterial hypertension and type 2 diabetes [41]. Bardoxolone methyl has been studied in the BEACON trial, where it showed significant improvements in kidney function for patients with stage 4 CKD. Although the trial was halted due to concerns about heart-related side effects, subsequent studies have focused on optimizing dosing and patient selection to mitigate these risks [41]. Moreover, the CARDINAL trial has explored its use in Alport syndrome, a genetic condition leading to kidney disease, with positive interim results suggesting improvements in kidney function and overall health [41,207]. The potential of bardoxolone methyl extends beyond kidney-related diseases. Its broad-spectrum anti-inflammatory and antioxidant effects are being researched for applications in various chronic inflammatory and oxidative stress-related conditions, positioning it as a promising therapeutic agent across multiple domains.

Oltipraz, originally developed by Rhône-Poulenc as a treatment for schistosomiasis, showed high cure rates exceeding 90% in field trials during the early 1980s. However, side effects, including digestive issues and fingertip pain exacerbated by sunlight, led to its abandonment in favor of less problematic treatments [41]. Oltipraz significantly reduced GSH stores in *Schistosoma mansoni* parasites while increasing GSH levels in host tissues. These findings indicated that oltipraz could induce enzymes that maintain reduced GSH pools and detoxify electrophiles, suggesting potential cancer chemopreventive properties at lower doses [41].

Sulforaphane, a highly reactive isothiocyanate, is produced from the inert precursor glucoraphanin found in broccoli plants, particularly in the aerial portions and seeds. When plant tissue is disrupted, myrosinase catalyzes the hydrolysis of glucoraphanin to yield sulforaphane, a process also facilitated by β-thioglucosidases in the human microbiome. Since 1998, over 75 studies have examined the pharmacokinetics, pharmacodynamics, and efficacy of sulforaphane using various broccoli-based formulations, leading to inconsistent dosing and confusion in results interpretation [41]. Despite promising preclinical findings, relatively few clinical studies have focused on the efficacy of sulforaphane against disease endpoints, though trials have explored its potential benefits in conditions like autism, schizophrenia, cardiovascular disease, and diabetes [41]. Clinical trials have demonstrated that sulforaphane can improve markers of oxidative stress and inflammation in patients with chronic diseases such as diabetes and chronic obstructive pulmonary disease [41].

Similarly, curcumin, the active compound in turmeric, has been extensively researched for its anti-inflammatory and antioxidant properties. One study showed that curcumin supplementation increased NQO-1 levels and reduced plasma malondialdehyde levels in patients with type 2 diabetes, indicating enhanced antioxidant activity via Nrf2 activation, significantly improving oxidative stress markers and overall metabolic profiles [41]. Another trial, however, found no significant effects of curcumin on Nrf2 target gene activities in patients with diabetic proteinuric CKD, highlighting the variability in responses [41].

These findings highlight the therapeutic potential of Nrf2 activators not only in neurodegenerative and inflammatory diseases but also in neuropathic pain management [19]. By enhancing Nrf2 activity, these compounds help reduce oxidative damage and inflammation, suggesting that Nrf2 modulation could be a key strategy in developing more effective treatments for neuropathic pain [21]. However, more extensive clinical trials are necessary to establish their effectiveness and safety. The potential for Nrf2-based therapies is significant, but comprehensive clinical validation is required to integrate these treatments into routine medical practice.

## 9. Future Directions and Conclusions

Future research should focus on elucidating the detailed molecular mechanisms of Nrf2 in neuropathic pain and identifying the most effective modulators [19]. Studies could investigate how Nrf2 interacts with specific cellular pathways in different types of neuropathic pain, such as diabetic neuropathy or chemotherapy-induced peripheral neuropathy [22]. Techniques like CRISPR-Cas9 could be used to edit genes and observe how changes in Nrf2 expression affect pain perception and inflammatory responses [208]. Additionally, identifying small molecules or natural compounds that can modulate Nrf2 activity with high specificity could lead to new therapeutic options [22]. For example, plant-derived compounds like sulforaphane have shown potential in activating Nrf2, and further research could optimize these compounds for clinical use [209].

Combining Nrf2 modulators with other therapeutic approaches, such as anti-inflammatory drugs or neuroprotective agents, could enhance their efficacy [210]. For example, a combination therapy involving an Nrf2 activator and an anti-inflammatory drug like ibuprofen could be tested in animal models of neuropathic pain to assess synergistic effects [19]. Similarly, the co-administration of Nrf2 modulators with neuroprotective agents, such as brain-derived neurotrophic factor mimetics, could be explored to see if they provide better pain relief and neuronal protection compared to single-agent therapies [38]. Clinical trials could be designed to evaluate these combinations in patients with chronic neuropathic pain conditions.

Personalized medicine approaches, considering individual genetic and environmental factors, may optimize treatment outcomes. For instance, the genetic profiling of patients could identify polymorphisms in the Nrf2 gene that affect its function or expression [211]. Patients with certain genetic variants might respond better to specific Nrf2 modulators, allowing for tailored treatment plans. Environmental factors, such as diet and exposure to toxins, could also influence Nrf2 activity [212]. Researchers could study how these factors interact with Nrf2 modulators to personalize treatments further. For example, a diet rich in Nrf2-activating foods might complement pharmacological treatments in certain patient populations [211].

Additionally, developing non-invasive biomarkers to monitor Nrf2 activation and therapeutic response will be crucial for advancing clinical applications [41]. Researchers could focus on identifying biomarkers in blood or saliva that correlate with Nrf2 activation in neural tissues [213]. These biomarkers could then be validated in clinical trials to ensure they accurately reflect therapeutic responses. Non-invasive imaging techniques, such as positron emission tomography scans with Nrf2-specific tracers, could also be developed to monitor the effects of treatments in real time [41,213].

Together, these strategies hold the potential to improve the management of neuropathic pain, offering new possibilities for effective and individualized patient care.

## Figures and Tables

**Table 1:** Nrf2-related activity of common drugs used to alleviate neuropathic pain.

Compound	Experimental Model	Dose/Concentration	Possible Mechanism	Ref.
Gabapentin	Sepsis-induced acute kidney injury in rats	50 and 100 mg/kg i.p. for 4 days	Upregulation of Nrf2/HO-1 pathway, suppressed apoptosis, attenuated oxidative stress	[129]
Pregabalin	Streptozotocin-induced diabetic mice	10 mg/kg i.p.	Higher expression of Nrf2 in brain, immunomodulation, suppression of inflammation	[130]
Amitriptyline	HUVEC cells	2.5 µM for 24 h	Alleviated inflammation due to suppressed NF-κB activity	[131]
Duloxetine	Rat mild chronic stress model	15 mg/kg i.p. for 7 days	Increased expression of Nrf2 and antioxidant enzymes in prefrontal cortex.	[132]
	Asthmatic BALB/c mice model	30 mg/kg i.g. for 10 days	Decreased inflammation due to Nrf2/HO-1 pathway activation in lungs	[133]
	Human neuroblastoma SH-SY5Y cells	1–5 µM for 24 h	Neuroprotective activity against oxidative stress and cell death via Akt/Nfr2/HO-1 pathway, nuclear translocation of Nrf2	[134]
Lidocaine	Lung A549 cells	0.5–10 mM	Attenuation of inflammation, apoptosis and ferroptosis due to regulation of p38 MAPK signaling pathway	[135]
	Murine allergic rhinitis model	1, 3, 5 mg/kg intranasally for 7 days	Reduced inflammation, suppressed NF-κB and p38 MAPK signaling pathways	[136]
Ketamine	Mixed-granulocyte murine asthma model	75 mg/kg i.p. for 4 days	Reduced inflammation due to activation of Nrf2/Keap-1 signaling pathway	[137]
	Experimental traumatic brain injury in mice	30, 60, 100 mg/kg i.p.	Increased Nrf2 level and induced expression of Nrf2 signaling pathway-related proteins	[138]
	Lipopolysaccharide-stimulated macrophages	100–1000 µM for 12 h	Activated Nrf2/HO-1 signaling pathway, suppressed NF-κB.	[139]

**Table 2:** Nrf2-related activity of alternative/emerging drugs used to alleviate neuropathic pain.

Compound	Experimental Model	Dose/Concentration	Possible Mechanism	Ref.
Ambroxol	Acetic acid rat model of ulcerative colitis	100, 200 mg/kg per os for 8 days	Enhanced Nrf2 activity, upregulated HO-1 and catalase, inactivated NF-κB signaling, suppressed IL-6 and TNF-α.	[155]
	Mouse macrophages (RAW 264.7) Human keratinocytes (HaCaT) cell line	7.8 to 500 μM	Decreased the protein expression of nitrotyrosine, iNOS, NF-κB and MAPKs signaling cascade, increases expression of Nrf-2 and SOD-1 in RAW 264.7 cells and skin tissue	[156]
	Psoriasis-like BALB/c mice skin inflammation model	10, 30 mg/kg/day topically for 6 days		
Cannabidiol	Lipopolysaccharide-induced rat neuroinflammation model	5 mg/kg i.p.	Suppressed neuroinflammation via regulating AKT, CREB, BDNF expressions, Nrf2 signaling, apelin and tyrosine hydroxylase synthesis.	[157]
	Gentamicin-induced rat nephrotoxicity	2.5, 5, and 10 mg/kg/day for 10 days	Activation of the FXR/Nrf2 pathway	[158]
	Paclitaxel-induced murine peripheral neuropathy	10 mg/kg i.p. twice a week for six weeks	Restored p-AMPK, SIRT1, Nrf2, HO-1, SOD2 levels	[159]
Bromelain	Rat chronic constriction injury	30, 50 mg/kg per os	Nrf2 activation and translocation to nucleus	[160]
Melatonin	Rat myenteric neuron damage during colitis model	2.5 mg/kg i.p.	Alleviated inflammation and oxidative stress via increased the Nrf2 and HO-1 levels.	[161]
	ApoE^−/−^ mice	20 mg/kg/day i.p.	Inhibited macrophage ferroptosis due to activation of Nrf2 pathway	[162]
	Severe acute pancreatitis murine model	5, 20 50 mg/kg i.p.	Protected from multiorgan damage via increased Nrf2 expression	[163]
*N*-acetyl-L-cysteine	Rat chronic prostatitis model	300 mg/kg i.p.	Activated the Keap1/Nrf2 signaling	[164]
Acetyl-L-carnitine	Rat hypoxia model	75 mg/kg per os	Mitochondrial biogenesis via ERK-Nrf2 mediated pathway	[165]
	Rat hypobaric hypoxia model	50 mg/kg i.p.	Reduced oxidative damage via ERK-Nrf2 mediated pathway	[166]
Palmitoylethanol-amide	Rat spinal cord injury model	2 mg/kg i.p.	Inhibited inflammatory responses and oxidative stress, due to activation of Nrf2/HO-1 pathway	[167]
Resveratrol	Rat chronic constriction injury	20 mg/kg, per os	Activation of Nrf2 and antioxidant enzymes	[168]
	Mice diabetic peripheral neuropathy	10% 10 mL/kg, i.g.	Suppression of the expression of NF-κB by upregulating the activation of Nrf2 and the expression of antioxidant enzymes	[169]
	Rat chemotherapy-induced peripheral neuropathic pain	6.28 and 7.81 mg/kg/day p.o.	NF-κB inhibition, Nrf2 activation	[170]
Curcumin	Oxaliplatin-induced neuropathic pain mouse model	10 mg/kg i.p. for 7 days	Inhibited the NLRP3 inflammasome-mediated inflammatory response, increased Nrf2/GPx4-mediated antioxidant responses, and decreased mitochondrial oxidative generation	[71]
	Knee osteoarthritis rat model	100 μg/mL 100 μL injection into a knee cavity 2× week for 8 weeks	Antioxidant activity via Nrf2/HO-1 signaling pathway	[171]
Sulforaphane	Human nucleus pulposus tissue Rat intervertebral disc degeneration model	10 μM for 24 h 10 μmol/L intradiscal injection every second week for 8 weeks	Delayed intervertebral disc degeneration by alleviating endoplasmic reticulum stress in nucleus pulposus cells via activating Nrf2/HO-1	[172]
	Human eccentric exercise model	30 mg/day for 7 days	Increased *NQO1* mRNA expression in peripheral blood mononuclear cells	[173]
	Rat cancer-induced bone pain model	0.1, 0.5, 1, 5 or 10 mg/kg injected intrathecally for 7 days	Alleviated cancer pain via Nrf 2, HO-1 activation, NF-κB inhibition, inflammatory marker suppression. Also enhanced pain relief by morphine.	[174]
	Rat sciatic endo metriosis	5, 15, 30 and 60 mg/kg, i.p. for 28 days	Keap1/Nrf 2 pathway upregulation, suppression of inflammation and oxidative stress	[175]
	CCI-induced murine neuropathic pain	10 mg/kg, i.p. for 7 days	Nrf 2/HO-1/NQO-pathway activation, microglial activation, MAPK phosphorylation	[176]

**Table 3:** Clinical effects of Nrf2 modulators.

Compound	Developer/Trial	Original Use	Effects	Ref.
Dimethyl fumarate	Fumaderm^®^ (Biogen, Cambridge, MA, USA)	In combination with mono ethylfumarate—for moderate to severe plaque psoriasis	Prevents degradation of Nrf2 activity, enhances expression of genes protecting from inflammation and oxidative damage.	[206]
	Skilarence^®^ (Almirall Limited, Uxbridge, Middlesex, UK)	For moderate to severe plaque psoriasis		
	BG-12, Tecfidera^®^ (Biogen, Cambridge, MA, USA)	Approved for relapsing-remitting multiple sclerosis		
Bardoxolone methyl	BEACON trial	For treatment of chronic kidney disease and other inflammatory conditions	Nrf2 inducer with broad-spectrum anti-inflammatory and antioxidant effects	[207]
	CARDINAL trial	For use in Alport syndrome		
Oltipraz	Developed by Rhône-Poulenc (Vitry-sur-Seine, France)	For schistosomiasis	Could induce enzymes that maintain reduced GSH pools and detoxify electrophiles	[41]
Sulforaphane	Few clinical studies on autism, schizophrenia, cardiovascular disease, and diabetes	n.d.	Can improve markers of oxidative stress and inflammation in patients with chronic diseases	[41]

## Data Availability

All data are included within the manuscript.
